# Schizophrenia: What’s Arc Got to Do with It?

**DOI:** 10.3389/fnbeh.2017.00181

**Published:** 2017-09-20

**Authors:** Francesca Managò, Francesco Papaleo

**Affiliations:** Department of Neuroscience and Brain Technologies, Istituto Italiano di Tecnologia Genova, Italy

**Keywords:** behavior, RDoC, dopamine, glutamate, immediate early gene, Arg3.1, mice

## Abstract

Human studies of schizophrenia are now reporting a previously unidentified genetic convergence on postsynaptic signaling complexes such as the activity-regulated cytoskeletal-associated (Arc) gene. However, because this evidence is still very recent, the neurobiological implication of Arc in schizophrenia is still scattered and unrecognized. Here, we first review current and developing findings connecting Arc in schizophrenia. We then highlight recent and previous findings from preclinical mouse models that elucidate how Arc genetic modifications might recapitulate schizophrenia-relevant behavioral phenotypes following the novel Research Domain Criteria (RDoC) framework. Building on this, we finally compare and evaluate several lines of evidence demonstrating that Arc genetics can alter both glutamatergic and dopaminergic systems in a very selective way, again consistent with molecular alterations characteristic of schizophrenia. Despite being only initial, accumulating and compelling data are showing that Arc might be one of the primary biological players in schizophrenia. Synaptic plasticity alterations in the genetic architecture of psychiatric disorders might be a rule, not an exception. Thus, we anticipate that additional evidence will soon emerge to clarify the Arc-dependent mechanisms involved in the psychiatric-related dysfunctional behavior.

## Schizophrenia

Neuropsychiatric disorders are still defined by an ensemble of different behavioral abnormalities appearing with a certain intensity and duration that strongly disrupt the normal life of the affected individuals (American Psychiatric Association, 2013). However, these behavioral alterations present huge heterogeneity within and between subjects in terms of intensity, timing, penetrance depending on the sex and age of the subjects as well as assessment by different health care providers and responses to treatments. “Schizophrenia spectrum and other psychotic disorders” is a definition given when clinical features categorized as positive (e.g., delusions, hallucinations, disorganized thinking, grossly disorganized or abnormal motor behavior) and negative symptoms (e.g., diminished emotional expression and avolition) are evident in an individual (Lewis and Gonzalez-Burgos, 2006; American Psychiatric Association, 2013). In addition, cognitive deficits, mainly in executive functions, are long-lasting traits in patients diagnosed with schizophrenia and constitute key prognostic factors for the long-term outcomes of the disease such as the level of functional capability, social and occupational ability and quality of life (Green, 1996; Green and Nuechterlein, 1999; Mueser and McGurk, 2004). Thus, it is clear that such a complex disorder uniquely defined by several different behavioral abnormalities is subject to different degrees of heterogeneity.

Consistent evidence indicates that many psychiatric disorders such as schizophrenia have a strong genetic contributing factor with heritability estimated at up to 80% (Cardno and Gottesman, 2000; Sullivan et al., 2003). Because of this, over the last decade, an increasing effort has been made to disentangle the possible impact of genetics in the development and heterogeneity of schizophrenia and of psychiatric disorders in general (Fromer et al., 2014; Purcell et al., 2014; O’Donovan and Owen, 2016). Despite other factors being implicated (e.g., environmental, epigenetics etc.), the current hope in advanced genetic assessments is to improve the causal understanding of psychiatric disorders, to provide a better definition of them, and to ultimately identify better and more effective treatments.

## Arc Genetics in Schizophrenia

Thanks to recent improvements in genomic sequencing techniques, Fromer et al. (2014) were able to run an exome sequencing study scanning genes for the *de novo* mutation at the single-base resolution on genomic DNA of 623 schizophrenia proband trios. Furthermore, Purcell et al. (2014), focusing on a subset of almost 2500 genes that have been previously implicated in schizophrenia, have sequenced the currently largest sample of patients with schizophrenia (2536) and healthy controls (2543). Both these works have unexpectedly revealed a consistent convergence of genetic variations on a set of synaptic proteins that interact with the activity-regulated cytoskeleton associated protein (Arc; Fromer et al., 2014; Purcell et al., 2014). For example, among the 28 genes listed in the “Arc complex”, it has been proven that Arc protein directly binds Wave1, GKAP, IQSEC2, GluNA2 (Myrum et al., 2015a; Zhang et al., 2015). However, further biochemical experiments should be performed to substantiate and better validate the mechanisms of interaction within this Arc complex. Nevertheless, in the same year, another study reported reduced expression of Arc mRNA in the prefrontal cortex (PFC) of individuals with schizophrenia (Guillozet-Bongaarts et al., 2014). Moreover, an investigation on variants within the Arc gene reported a direct association between the rs35900184 single-nucleotide polymorphism (SNP) and schizophrenia (Huentelman et al., 2015). To further support the possible implication of Arc in the pathophysiology of schizophrenia, the copy number variance (CNV) and schizophrenia working groups of psychiatric genomics consortium analyzed the CNV of the entire genome comparing more than 40,000 individuals among patients and healthy controls. Again, deletions in a subset of genes belonging to the Arc complex were significantly enriched in patients with schizophrenia (Marshall et al., 2017). Finally, different mouse models relevant to schizophrenia show reduced Arc expression, which is instead not evident in mouse models relevant to other psychiatric disorders such as ADHD or bipolar disorders (Matsuo et al., 2009; Takao et al., 2013; Takagi et al., 2015; Managò et al., 2016; Chen et al., 2017; Mereu et al., 2017). This might suggest that the Arc pathway may serve as a hub that functionally links numerous schizophrenia risk-related factors. Together, these findings point to a previously undetected association of the immediate early gene (IEG) Arc to schizophrenia. However, Arc causal implication in the neuropathophysiology of schizophrenia is just starting to be elucidated (following paragraphs).

## Arc Genetics in Behavior

Psychiatric disorders, including schizophrenia, are characterized by abnormal behaviors. Thus, here we will first address the implication of Arc genetics in behavioral functions. In particular, we will follow the new Research Domain Criteria (RDoC) framework recently developed by the USA National Institutes of Health (NIH/NIMH, 2017). The RDoC system currently includes five distinct domains: (1) Cognitive Systems. (2) Systems for Social Processes. (3) Positive Valence System. (4) Negative Valence Systems. (5) Arousal/Regulatory Systems). The RDoC framework aim to integrate many levels of information (from genomics to self-report) with specific dimensions of behavioral functioning, overcoming the boundaries of mental diagnosis. Indeed, within a disease as defined by DSM-V, alterations of different brain circuits or neurotransmitters could affect the same behavior. Alternatively, in different psychiatric illnesses, the same biological alteration could lead to a common behavioral alteration.

To our knowledge, there are still no selective Arc genetic variations in humans proven to be functional (i.e., altering Arc mRNA and/or protein expression). Only one study reported a case of a 7-year old female with a 540 kb microdeletion in the 8q24.3 region, which included Arc but also several other genes (Hu et al., 2015). This patient showed developmental abnormalities, Intellectual Disabilities (ID), autism and attention deficit hyperactive disorder (ADHD). Similarly, the mother, who carried the same microdeletion, presented a milder phenotype, including learning disabilities, depression, panic disorder and obsessive tendencies (Hu et al., 2015). However, this microdeletion syndrome does not account for the selective impact of Arc genetic disruption in behavioral abnormalities. Due to the lack of human data on functional common genetic alterations selective for Arc, our discussion will be centered on the available information derived from preclinical studies addressing the impact of selective Arc functional genetic variations in behavioral functioning.

## Cognitive Systems

Arc genetic variations were initially implicated in the formation of long-term memories. Indeed, compared to wild-type mice, Arc knockouts performed slightly worse in the spatial Morris water maze, were impaired in contextual and cued fear conditioning, showed reduced conditioned taste aversion, and impaired long-term novel object recognition memory (Plath et al., 2006). All these abnormal cognitive functions suggest that reduced levels of Arc might be related to hippocampus-dependent memory deficits. Indeed, blocking the expression of Arc selectively in the hippocampus produced the same pattern of performance in the above mentioned tasks, including reduced spatial and fear memory formation in the Morris water maze and fear conditioning task (Guzowski et al., 2000; Chia and Otto, 2013; Nakayama et al., 2015). Altered long-term memories might be present in patients with schizophrenia (Goldberg et al., 1989; Aleman et al., 1999; Ranganath et al., 2008), even if this is not considered a signature feature. For example, patients with schizophrenia might present episodic memory deficits due to an altered pattern of hippocampal-PFC activity, but they do not show an amnesic syndrome (Ranganath et al., 2008). More recently, we found that partial and complete deletion of the Arc gene in mice produced recency memory deficits in the temporal order object recognition task as well as spatial memory deficits in the spatial object recognition task (Managò et al., 2016). In contrast, cognitive abilities assessed by a recent-memory novel object recognition task reported to be dependent uniquely on the perirhinal cortex (PRH; Barker et al., 2007) were intact (Managò et al., 2016). These findings parallel similar evidence from patients with schizophrenia who show impairments in temporal context memory related to objects as well as in spatial navigation, while no alterations are evident in the ability to recall and recognize target items (Schwartz et al., 1991; Rizzo et al., 1996; Dreher et al., 2001; Folley et al., 2010). These recent mouse studies (Managò et al., 2016) begin to suggest that Arc-dependent cognitive abnormalities might rely on altered PFC and hippocampal dysfunction in the context of a normal functioning of the PRH. In agreement, convergent genetic, molecular, clinical, neurophysiological, neuropsychological and imaging work confirmed the presence of an altered pattern of PFC and hippocampal activation in schizophrenia (Meyer-Lindenberg and Weinberger, 2006; Papaleo et al., 2012; Millan et al., 2014). Initial work did not find an association between Arc common genetic variants and general cognitive abilities in healthy subjects (Myrum et al., 2015b). However, the Arc genetic variations investigated were not shown to have a functional impact on Arc protein or mRNA expression. Thus, future studies will be needed in order to disentangle the selective implication of Arc functional genetic variations in working memory performance and executive functions, the two cognitive domains at the basis of schizophrenia neuropathophysiology.

## Systems for Social Processes

In recent years, there has been growing consensus that abnormalities in social cognition form part of the core symptoms in schizophrenia (Billeke and Aboitiz, 2013; Millan et al., 2014). Individuals with schizophrenia have marked impairments in processing non-verbal social affective information while showing normal affect sharing and emotion experience (Green et al., 2015). Notably, social cognitive impairments in these individuals have a more deleterious impact on daily functioning than non-social cognitive deficits (Fett et al., 2011). Arc knockout mice show impaired social abilities as demonstrated by reduced sociability and reduced preference for social novelty (Managò et al., 2016). In particular, in the 3-chamber paradigm, Arc knockout mice preferred to be in the chamber with an empty cup rather than with a novel conspecific. Moreover, Arc knockout mice were not able to discriminate between a novel and a familiar conspecific. These social measures were obtained in a well-established test for mice used to assess social avoidance and preference for social novelty (Moy et al., 2004, 2008). Decreased interaction with conspecifics is an index of social withdrawal reminiscent of what is observed in patients with schizophrenia. Indeed, low social reciprocity with others and deficits in social cognition (Harvey et al., 2006) are core features of schizophrenia negative symptomatology. Moreover, these symptoms are also enduring and less responsive to medication, not to mention among the most disabling features of this psychiatric illness. Therefore, the reduced sociability and preference for social novelty shown by Arc knockout mice is consistent with the deficits seen in patients with schizophrenia and represent further evidence supporting the role of reduced Arc levels in schizophrenia neuropathology. However, we should highlight that despite their extensive use and importance, currently available tasks assessing social functions in rodents are still limited in their equivalence to tasks used in the human clinical setting. Indeed, social cognitive processes such as theory of mind, facial perception/recognition, and emotion regulation are the social processes mostly impaired in schizophrenia (Green et al., 2015). These social cognitive functions are not yet directly and specifically testable in rodents. This will require consistent efforts in the field with a clear aim to prove the predictive translational validity of novel and more refined social cognitive tasks in rodents.

## Positive Valence System

This domain involves processes such as motivation, responsiveness to reward and habit formation. In schizophrenia, the hedonic responses to reward and willingness to work for a reward (motivational state) are impaired (Gard et al., 2009). Unfortunately, as far as we know, there is little evidence demonstrating that genetic variations in Arc play a role in these processes. However, recent work has begun to address this domain. One study reported that Arc knockout mice develop a cocaine-conditioned place preference (Salery et al., 2017), at doses that are ineffective in wild-type mice (Contarino et al., 2017). This suggest that Arc genetic disruption might increase rewarding effects of psychostimulant drugs, but further work will be needed in this novel and interesting area of research.

## Negative Valence Systems

No alterations in anxiety-like states have been found in Arc knockout mice as measured by the O-maze and light-dark tests (Plath et al., 2006). Similarly, reactivity to acute threats such as mild foot shocks (Plath et al., 2006) or sudden acoustic sensory stimuli (Managò et al., 2016) was not altered in Arc knockout mice. However, overall Arc genetic disruption as well as knocking down Arc expression selectively in the lateral amygdala was enough to produce a deficit in fear conditioning memories (Ploski et al., 2008). Thus, these data suggest a marginal role of Arc genetic variations in the negative valence domain, with more direct involvement in the storage and expression of aversive memories.

## Arousal/Regulatory Systems

This domain includes processes responsible for generating activation of neural systems as appropriate for various contexts, and providing appropriate homeostatic regulation (subcategories: arousal, circadian rhythms and sleep/wakefulness). Arousal represent the time of perception of internal/external stimuli related to the coding of relevant vs. non-relevant stimuli of the environment. Hippocampal CA1 recordings of local field potential during locomotion revealed a reduced power in the gamma and beta-2 range in Arc knockout mice compared to wild-type, indicating a disruption in the neuronal synchronization during active behavior (Malkki et al., 2016). In agreement, Arc knockout mice show altered activity when exposed to a newly-presented environment. In particular, both a slightly hyperactive (Managò et al., 2016) or hypoactive (Salery et al., 2017) phenotype have been reported. However, it is worth noting that the experimental setting of the latter study might have produced misleading and less sensitive data in locomotor activity as it was based on the breaking of only four beams placed at 90 degree points of a circular corridor. More consistent instead, Arc knockout mice showed increased locomotor sensitivity to dopaminergic psychostimulants including amphetamine (Managò et al., 2016) and cocaine (Salery et al., 2017). Moreover, repeated exposure to amphetamine produce, in the dorsal striatum and nucleus accumbens, a selective increase in a subset of mRNAs including Arc (Biever et al., 2017). Finally, the psychostimulant-induced increase in Arc expression seems to be evident mostly in D1-positive medium spiny neurons as well as in NMDA-positive neurons in striatal regions (Biever et al., 2017; Salery et al., 2017). Overall, these evidence point to Arc as an integrator of D1 and NMDA signaling and demonstrate that Arc genetic disruption causes a predisposition to higher sensitivity to psychostimulants.

Psychostimulant super-sensitivity is used as a rodent correlate of schizophrenia-like positive symptoms (Arguello and Gogos, 2006; van den Buuse, 2010) and is relevant to the arousal domain of the RDoC system. In particular, amphetamine exacerbates psychotic experiences in patients with schizophrenia and can be psychotogenic in normal subjects (Laruelle et al., 1999). Thus, Arc knockout mutants’ locomotor activity phenotypes are consistent with an increased arousal state to external stimuli and might be seen as a proxy of schizophrenia-like positive symptoms. Possibly due to different arousal states, Arc knockout mice also show prepulse-inhibition (PPI) deficits (Managò et al., 2016). PPI is considered a measure of sensorimotor gating consistently conserved from rodents to humans (Braff and Geyer, 1990). There have been numerous reports of PPI deficits in patients with schizophrenia (Swerdlow et al., 2008), their unaffected first degree relatives (Cadenhead et al., 1993, 2000), and patients with schizotypal personality disorder (Cadenhead et al., 1993). Thus, the PPI deficits in Arc knockout mice are consistent with a schizophrenia-relevant behavioral endophenotype.

Related to sleep processes instead, initial studies reported that Arc knockout mice do not show any differences in the composition of sleep (Malkki et al., 2016). This suggest a marginal implication of Arc genetics in relationship to sleep and wakefulness, and a negligible implication of “off-line” processing (e.g., during post-behavioral sleep) in cognitive deficits.

## Arc Biology

Arc is only present in Ca^2+^/Calmodulin-dependent kinase II alpha (CaMKIIa) expressing neurons in the hippocampus, neocortex and striatum (Vazdarjanova et al., 2006; Miyashita et al., 2008). Its expression is tightly regulated. Indeed, after a novel experience, Arc mRNA moves to the dendrites in the active synapse where is translated (Link et al., 1995; Lyford et al., 1995; Jakkamsetti et al., 2013). Here, Arc protein plays a critical role in long-lasting forms of synaptic plasticity, including long-term potentiation (LTP), long-term depression (LTD) and homeostatic scaling (Plath et al., 2006; Rial Verde et al., 2006; Shepherd et al., 2006; Park et al., 2008; Waung et al., 2008; Jakkamsetti et al., 2013). Thus, Arc might be considered as an integrator of different inputs coming from the nervous system in order to lead to a proper synaptic connection. In particular, Arc might work as a downstream regulator, and functional Arc genetic variations might represent a direct genetic bridge between different schizophrenia-related signaling systems. In this context, we will discuss possible molecular mechanisms of Arc in the modulation of glutamatergic and dopaminergic pathways, two systems extensively implicated in the schizophrenia neuropathology.

## Arc and Glutamate

Arc has been consistently linked to the glutamatergic system and reduced Arc protein expression alter glutamate-mediated processes such as learning and memory formation, cognition and neuronal plasticity (Guzowski et al., 2000; Park et al., 2008; Jakkamsetti et al., 2013; Wang et al., 2016). In particular, when Arc protein translation is disrupted, a high-frequency burst in the hippocampus is able to induce LTP; however, the second phase of consolidation of synaptic LTP is disrupted (Guzowski et al., 2000). In agreement, Arc has a fundamental role in the stabilization of actin filament at the synaptic site (Messaoudi et al., 2007). Moreover, Arc is implicated in the synaptic scaling of AMPA receptors for the induction of LTD, interacting with dynamin and endophilin (Chowdhury et al., 2006). In particular, Arc facilitates the endocytosis of AMPA receptor, a process that is implicated in the induction of LTD (Chowdhury et al., 2006; Shepherd et al., 2006). Notably, Arc can accumulate also at the inactive synapses binding to the inactive form of CamKIIbeta, consequently leading to the endocytosis of AMPA receptors (Okuno et al., 2012). Arc-dependent synaptic plasticity (LTP and LTD) is induced by the activation of mGluR1 or R5 (mGluR type I; Park et al., 2008; Kumar et al., 2012; Wang et al., 2016), and requires the involvement of eEF2 and FMRP that are implicated in the translation of Arc mRNA to protein (Park et al., 2008; Wang et al., 2016). However, despite the established involvement of Arc in mGluR-dependent plasticity (Park et al., 2008; Waung et al., 2008), its role in NMDA-dependent plasticity is still controversial. For instance, the localization of Arc mRNA at active synapses on the dendrites requires NMDA activation (Steward and Worley, 2001; Bloomer et al., 2008). Furthermore, consolidation of memories leads to an increased Arc protein level (Guzowski et al., 2000; McIntyre et al., 2005), and blocking NMDA receptor reduced Arc expression induced by a learning process (Czerniawski et al., 2011). However, other evidence indicate that NMDA-induced LTP or LTD is Arc-independent (Park et al., 2008; Waung et al., 2008). More recently, Arc was involved in experience-induced cortical firing patterns correlated with Arc-dependent increase of NMDA activity (Ren et al., 2014). Overall, these findings highlight the importance of Arc in the consolidation of some types of NMDA-dependent memory formation. Thus, when Arc functioning is diminished, NMDA-dependent signaling is expected to be partially disrupted.

The glutamatergic system has been often implicated in the manifestation of schizophrenia-relevant clinical symptoms. Noncompetitive NMDA/glutamate receptor antagonists such as PCP, ketamine or MK801 have psychomimetic effects (Halberstadt, 1995; Andiné et al., 1999; Frohlich and Van Horn, 2014) reproducing many behavioral alterations reminiscent of positive, negative and cognitive symptoms of schizophrenia in healthy humans and exaggerating positive and negative symptoms in patients with schizophrenia (Coyle, 2006; Kantrowitz and Javitt, 2010). Moreover, from recent genome-wide association studies (GWAS), several genes belonging to the glutamatergic system were part of the 108 list of implicated loci (Schizophrenia Working Group of the Psychiatric Genomics Consortium, 2014). In particular, genes that encode subunits of NMDA and AMPA receptors were significantly coming out as being strongly implicated (Schizophrenia Working Group of the Psychiatric Genomics Consortium, 2014). In agreement, a number of pre-clinical studies in rodents reported that an alteration of NMDA or AMPA transmission might recapitulate different behavioral alterations in a number of RDoC domains that might possibly be related to schizophrenia-relevant endophenotypes (Wiedholz et al., 2008; Papaleo et al., 2012). Despite this, and the consequent remarkable effort of the academics and the industry, clinical results related to new treatments for schizophrenia targeting the glutamatergic system have been disappointing (Iwata et al., 2015; Bugarski-Kirola et al., 2016). In this context, Arc being a downstream effector of glutamatergic receptors, it might be a better target and a more consistent cause of the development of schizophrenia-relevant behavioral alterations.

## Arc and Dopamine

The long-standing pathophysiological hypothesis of schizophrenia involves a dysregulated dopaminergic system (Weinstein et al., 2017). In particular, the current hypothesis highlights that a hyperactive mesolimbic system through an aberrant activation of D2 receptors might be more related to the so-called “positive symptoms”. Instead, a hypoactive mesocortical dopaminergic system with a lower stimulation of D1 receptor in the PFC can lead to schizophrenia negative and cognitive symptoms (Winterer and Weinberger, 2004; Simpson et al., 2010; Slifstein et al., 2015). Notably, the most common first-line treatments for acute and chronic therapy for schizophrenia are antipsychotic drugs, all of which interact with dopamine/D2 receptors (D2) brain pathways (Miyamoto et al., 2005; Hasan et al., 2013). Finally, D2 receptors have been confirmed as one of the major schizophrenia-association genetic hits in the most recent GWAS studies (Schizophrenia Working Group of the Psychiatric Genomics Consortium, 2014).

Up until last year, there has been no evidence implicating Arc genetic variations as modulators of the dopaminergic system. Indeed, the only data available were those reporting changes in Arc expression induced by dopamine agonists or antagonists as just a marker for neuronal activity (Moro et al., 2007; Banerjee et al., 2009; Fumagalli et al., 2009). In contrast, we have now demonstrated that Arc genetic disruption result in selective alterations on different aspects of the dopaminergic system. In particular, Arc knockout mice have reduced amphetamine-induced dopamine release within the medial PFC (mPFC) and, in agreement, two-photon calcium imaging revealed a reduced mPFC activation following electrical stimulation of the ventral tegmental area (VTA; Managò et al., 2016). Treatment with a D1 agonist rescued the altered mPFC activity as well as recency memory deficits, demonstrating that the mPFC hypofunction was D1-dependent (Managò et al., 2016). Alternatively, infusing the D2 antagonist eticlopride directly into the nucleus accumbens of Arc knockout mice rescued their supersensitivity to amphetamine in terms of dopamine release and locomotor activity, unraveling a D2-dependent hyperactive dopaminergic mesolimbic system (Managò et al., 2016). These Arc-dependent effects were evident in the mPFC and in the nucleus accumbens, but not in the dorsal striatum. The source of dopamine in both the mPFC and nucleus accumbens is the VTA, while in the dorsal striatum it is the substantia nigra (Beckstead et al., 1979). Furthermore, amphetamine injection in Arc knockout mice produced opposing dopamine-release phenotypes in the mPFC compared to that in the nucleus accumbens. These contrasting effects in mesocortical and mesostriatal dopaminergic pathways might then suggest an Arc-dependent circuital dysfunction that will require further investigations. In conclusion, Arc function seems to be crucial for establishing a proper activity balance between mesocortical and mesostriatal dopaminergic circuits. Importantly, these alterations are reversible by selectively targeting D2 receptors in the ventral striatal regions and D1 receptors in the PFC.

Despite this previously unexpected evidence, the mechanisms underlying the peculiar effects of Arc genetics in the dopaminergic system are as yet unclear. Previous studies have reported that PFC dopaminergic inputs show protracted postnatal maturation through adolescence and are susceptible to activity-dependent modification during this period (Kalsbeek et al., 1988; Lewis and O’Donnell, 2000; Mastwal et al., 2014). Recurrent network activity in frontal-striatal loops can also affect striatal circuit maturation (Kozorovitskiy et al., 2012). As Arc protein is abundantly expressed in cortical excitatory and striatal GABAergic projection neurons (but not detected in midbrain dopamine neurons; Shepherd and Bear, 2011), it may regulate activity-dependent maturation of the VTA-PFC-striatal circuits during postnatal development. Considering the well-known role of Arc in modulating glutamate receptors (Shepherd and Bear, 2011; Jakkamsetti et al., 2013; Ren et al., 2014), and the balance between the glutamatergic and dopaminergic systems which tightly regulate each other, our recent findings raise the possibility that Arc-dependent changes in glutamatergic signaling might be the effector of the changes in the dopamine system. However, further studies are needed to unravel these issues and how Arc alterations at the single-cell level might affect these circuits.

## Beyond Schizophrenia

Findings from genetics studies might be applied to discrete behavioral domains (e.g., RDoC framework) overcoming the boundaries of psychiatric diagnosis. The current system for diagnosing psychiatric illnesses, based on DSM guidelines, relies on defining a constellation of signs and symptoms, each of which may be present in a number of different disorders, and none of which is, by itself, diagnostic. In support of this idea, recent findings indicate that different psychiatric disorders such as schizophrenia, autism, ADHD, intellectual disability and bipolar disorder, might share common genetic variations (McCarthy et al., 2014; Goes et al., 2016; Zhao and Nyholt, 2017). In this context, and because of its major modulatory impact in synaptic plasticity (Tzingounis and Nicoll, 2006; Bloomer et al., 2008; Park et al., 2008; Waung et al., 2008; Bramham et al., 2010; Gao et al., 2010; Ren et al., 2014; Wang et al., 2016), a role of Arc genetics in a number of different neurological and psychiatric disorders is not surprising (Greer et al., 2010; Cao et al., 2013; Ebert and Greenberg, 2013; Li et al., 2015). Indeed, Fromer et al. (2014) found that schizophrenia, autism spectrum disorder and ID share common genetic variations in the Arc complex. Despite this, to date, there have been no studies directly associating Arc genetic variations in other psychiatric disorders beyond schizophrenia. However, as already mentioned, one case with a rare microdeletion (8q24.3) encompassing the Arc gene demonstrated autistic traits, ID and ADHD (Hu et al., 2015). Moreover, genetic modifications associated with different syndromes such as the fragile X, Angelman and Autism Spectrum Disorder concern genes that encode for proteins involved in the regulation of Arc expression (Smith et al., 2011; Niere et al., 2012; Cao et al., 2013).

Patients with the Fragile X Syndrome (FXS) carry a triplet repeat expansion in the FMR1 gene that lead to reduced translation of the FMRP protein (Garber et al., 2008). The FMRP is a protein synthesis regulator and one of its targets is Arc (Park et al., 2008). In agreement, FMR1 knockout mice display higher production of Arc and consequent abnormal LTD (Niere et al., 2012; Ebert and Greenberg, 2013). The FXS is characterized by social impairments, cognitive disabilities, mood disorders and hyperactivity (Garber et al., 2008), which are all behavioral domains affected by Arc genetic variations (see above). Thus, it might be plausible that altered Arc expression is one of the causes of these behavioral abnormalities.

The Angelman Syndrome (AS) is caused by the deletion or inactivation of the maternal copy of the Ube3a gene (Williams et al., 2010). The *Ube3a* gene encodes for a brain-specific E3 ubiquitin ligase which has Arc as one of its substrates. In agreement, loss of Ube3A in mice cause an increase in Arc levels (Cao et al., 2013). The core symptoms of this pathology are delayed motor milestones, mental retardation, seizures, movement or balance disorders (Williams et al., 2010), once again asserting a potential implication of Arc-dependent mechanisms. Similarly, a genetic variation characterized by the appearance of Ube3A extra copies have also been associated with the autism spectrum disorder (Smith et al., 2011). Indeed, patients with this mutation present impaired social and communication deficits as well as repetitive behaviors (Smith et al., 2011; Bourgeron, 2015). Similarly, transgenic mice with three copies of the Ube3A manifest social deficits and increased self-grooming compared to the control group. Moreover, this mutation produced an impairment in the glutamatergic transmission and decreased Arc availability (Smith et al., 2011). Because these pathologies share common behavioral alterations in cognitive and social functions modulated by Arc genetics, we might hypothesize Arc as a converging downstream signaling output.

Finally, it seems that Arc could be involved also in Alzheimer’s disease (AD). Indeed, Arc can directly bind presenilin1 to regulate γ-secretase activity in order to form more β-amyloid peptides, participating in the formation of neuritic plaques. Furthermore, the same study has reported increased Arc protein levels in patients with AD (Wu et al., 2011). Despite the potential direct role of Arc in the formation of β-amyloid peptides, both increased (Wu et al., 2011) and decreased (Bi et al., 2017) Arc expression have been reported in the cortex of patients with AD. Moreover, initial GWAS on European and American subjects did not reveal any association between Arc genetic variation and AD (Lambert et al., 2013). Nonetheless, a more recent study has described a possible association of a SNP (rs10097505) in the 3’UTR of the Arc gene with susceptibility to AD (Bi et al., 2017). Thus, further work will be needed to understand the possible involvement of Arc genetics in the AD pathology and especially in its cognitive manifestations.

## Conclusions and Future Directions

**Figure 1:**
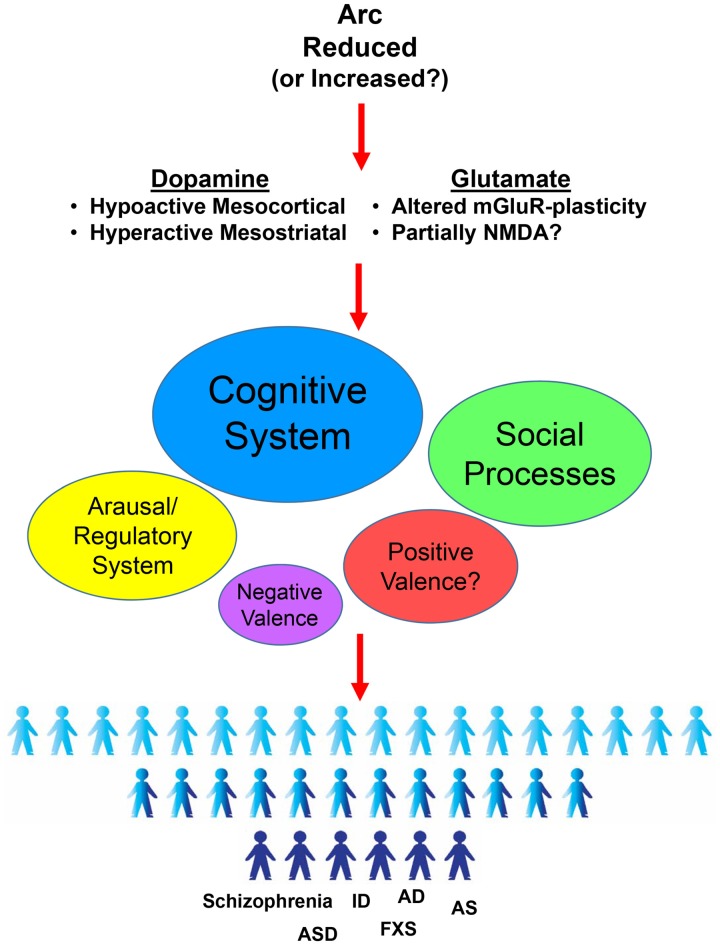
Activity-regulated cytoskeletal-associated (Arc)-dependent effects in Research Domain Criteria (RDoC) behavioral domains and its putative role in psychiatric disorders. Arc genetic disruption have been reported to alter both the dopaminergic and glutamatergic systems in a very selective way. This Arc-dependent altered neurotransmission results in deleterious effects in different behaviors. In particular, following the RDoC framework (NIH/NIMH, 2017), altered levels of Arc induce consistent impairments mainly in cognitive systems, but also in social processes and in arousal/regulatory systems. More investigations are needed for the positive valence system. No major influence seems to be evident for the negative valence system. Ultimately, Arc-dependent alterations in these behavioral processes might converge in a pathological state. In agreement, genetic variations suggested to alter Arc expression have been implicated in different diseases such as Schizophrenia (Schizophrenia), Autism (ASD), Intellectual Disabilities (ID), Fragile X Syndrome (FXS), Angelman Syndrome (AS) and Alzheimer’s Disease (AD).

The evidence discussed here highlight the consistent implication of Arc genetic variations in the development and manifestation of a number of behavioral abnormalities relevant to schizophrenia and other psychiatric disorders. In particular, mouse studies indicate a preponderant role of Arc in behavioral domains including cognitive, social and arousal processes, which might depend on the alterations of the glutamatergic and dopaminergic systems (Figure 1).

Despite this, it is still unknown how disruption of Arc can recapitulate so divergent and selective alterations in the dopaminergic system. For example, it is not clear if the cause of the dopamine system dysfunction is driven by Arc disruption of the glutamatergic signaling or if Arc might exert a direct influence on dopaminergic pathways. Furthermore, it is still unclear if Arc might play a role in behavior directly altering it or through developmental processes or both. This will be particularly relevant as early detection and early intervention of cognitive and social deficits could be potentially more effective in mitigating the pathological trajectories and ultimately the life quality of individuals with schizophrenia-vulnerability. In this context, mouse models will be useful tools in the development and testing of early diagnosis and early treatment strategies, at the same time strictly controlling for environmental and genetic factors. An aberrant maturation of the PFC has been reported in schizophrenia (Lewis and Levitt, 2002) and it is well known that the final maturation of dopaminergic terminals in the PFC is only reached after puberty (Manitt et al., 2011). Arc mRNA expression starts to increase after postnatal day 7 in the cortex, and its activation depends on the correct dopaminergic input coming from the VTA (Ye et al., 2016). Thus, we might hypothesize that this dopamine-induced Arc expression during postnatal development could be important for the correct establishment of synaptic connectivity within the mesocortical circuit. However, we cannot exclude an involvement of Arc in the prenatal developmental process as the presence of Arc in the brain has been detected since embryonic stages (Alberi et al., 2011). Identifying the developmental functions of Arc would also be relevant to other neurodevelopmental disorders such as autism, FXS and AS as discussed above. Therefore, studying the role of Arc in brain development will be important.

In conclusion, a concerted effort between clinical and preclinical genetic and mechanistic studies focused on Arc modulation of behavioral outputs looks to be a promising area of investigation. Indeed, this could greatly advance our knowledge on the causes of schizophrenia, especially in the areas of cognitive and social alterations. Notably, a better understanding of genetic variations that affect Arc, or its binding partners, might help to pave the way to more efficient treatments and prevention strategies in keeping with the promises of precision medicine. In particular, individual variability in Arc genetics could provide valuable tools to better address abnormalities in cognitive and social processes.

## Author Contributions

FP and FM found the materials and wrote the article.

## Conflict of Interest Statement

The authors declare that the research was conducted in the absence of any commercial or financial relationships that could be construed as a potential conflict of interest.
